# SO_2_ enhanced desorption from basic aluminum sulfate desulphurization–regeneration solution by falling-film evaporation

**DOI:** 10.1039/c7ra12963g

**Published:** 2018-02-01

**Authors:** Kuo Huang, Xianhe Deng, Feiqiang He

**Affiliations:** Department of Chemistry and Chemical Engineering, South China University of Technology Guangzhou Guangdong 510640 People's Republic of China huangkuo2006@126.com +86-020-87111814 +86-020-87111814; Guangzhou Institute of Energy Testing Guangzhou Guangdong 511447 People's Republic of China; School of Chemistry, Biology and Material Science, East China University of Technology Nanchang Jiangxi 330013 People's Republic of China

## Abstract

To find the optimal structure of the converging–diverging tube and develop a high-efficiency falling-film evaporator, the heat and mass transfer performances of falling-film evaporation with converging–diverging tubes of different dimensions were studied. The optimal converging–diverging tube was used in falling-film evaporation desorption of the basic aluminum sulfate desulphurization–regeneration solution, and different influential factors on the desorption effect were analyzed. It was found that converging–diverging tubes with large falling-film flow rate performed well in the heat and mass transfer of falling-film evaporation, and their rib height largely affected the heat and mass transfer performances. At the same rib height and rib pitch, the longer the converging segment of the converging–diverging tube was, the better the heat transfer performance was. The evaporation heat transfer coefficient and evaporation mass transfer rate in the optimal converging–diverging tube were 1.6 and 1.38 times larger than the smooth tube, respectively. The optimal converging–diverging tube was used in falling-film evaporation desorption of basic aluminum sulfate desulphurization–regeneration solution, at a perimeter flow rate of 0.114–0.222 kg m^−1^ s^−1^, the desorption efficiency inside the tube was up to 94.2%, which was 10.3–10.5% higher than that of the smooth tube. At the inlet sulfur concentration of 0.02–0.1 kmol m^−3^, the desorption efficiency was up to 94.1%, which was 12.0–16.3% larger than that of the smooth tube. At the heating temperature of 371.15–386.15 K, the desorption efficiency was up to 93.4%, which was 6.7–11.5% larger than that of the smooth tube. Smaller falling-film flow rate, higher sulfur concentration, or higher heating temperature was more constructive to SO_2_ desorption. Correlations were obtained to predict the mass transfer coefficient and SO_2_ desorption efficiency. This study develops a new type of falling-film evaporator for SO_2_ desorption from basic aluminum sulfate desulphurization–regeneration solution and provides a basis for process design and industrial application.

## Abstract 2

The heat and mass transfer performances of falling-film evaporation with converging–diverging tubes of different dimensions were studied.

## Graphical abstract

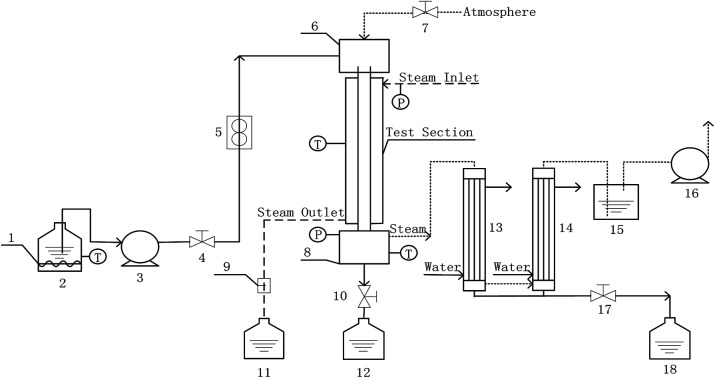

## 1. Introduction

Along with rapid socioeconomic development, great achievements have been made in flue gas desulfuration technology throughout the world. Statistics show that about 85% of existing flue gas desulfuration technology is wet technology, which has become the major technical trend of flue gas desulfuration.^1^ However, the nonrenewable wet desulfuration technology is limited by the nonrenewability of absorbents, large consumption, and generation of secondary waste, such as limestone suspension^2^ and seawater.^3^ Thus, the key to renewable wet desulfuration technology is to discover renewable desulfurization agents. The existing renewable desulfurization agents primarily include magnesia–magnesium sulfite, sodium citrate, organic amine, sodium sulfite, and basic aluminum sulfate. Research on the desulphurization method of some agents has been carried out and some defects have been found. As for magnesia–magnesium sulfite method, the calcination of magnesium sulfite into magnesia and SO_2_ requires temperatures up to 650–900 °C ^4^ and the byproduct magnesium sulfate can hardly be decomposed.^5^ In the sodium citrate method, sodium sulfite (or sodium bisulfite) can be easily oxidized into sodium sulfate, which will be separated out as crystals that block the equipment and tubes.^6^ In case of the organic amine method, despite its high efficiency of up to 95%, its desorption rate is rather low.^7^ In the strong sodium sulfite method, the large dosage of absorbent and the tendency of oxidation into sodium sulfate lead to its low desorption rate; also, sodium sulfate is hard to separate and even the separated sodium sulfate contains sodium sulfite crystals, which cause secondary pollution.^8^ In comparison, basic aluminum sulfate is very stable and can be prepared from cheap raw materials at low prices. Basic aluminum sulfate, as a promising desulfurization agent, has attracted wide attention from the research field. For instance, basic aluminum sulfate has been used to absorb SO_2_ from flue gas,^9–12^ which proves the high absorptive ability, and our team has carried out the mechanism research.^13^ However, research has been rarely conducted on desorption of the basic aluminum sulfate desulphurization–regeneration solution, which is a key step in the renewable wet desulfuration process. Thus far, water bath heating^14^ assisted by mechanical agitation,^15^ microwaves,^16^ ultrasonic waves^17–19^ or vacuum-pumping^20^ has been applied for enhanced desorption, exhibiting some improvements. However, these methods are limited by nonuniform heating and long desorption time; moreover, the relevant research is still at the laboratory stage, which is hard to industrialize. To overcome the above limitations and meet the requirements for industrialization, we find it necessary to adopt high-efficiency heat and mass transfer falling-film evaporator.

The converging–diverging tube has a periodic alternation of converging segments and diverging segments and thus, it exhibits enhanced heat and mass transfer performances.^21–24^ It has been used in various heat transfer facilities, such as condensers, air preheaters, waste heat boilers and oil coolers, and has been well-promoted in sulfuric acid, fertilizer, chemical and other industries.^25–27^ As an excellent enhanced heat transfer unit, the converging–diverging tube is primarily used for single phase liquid enhanced heat transfer inside and outside the tube,^28,29^ but has not been used in falling-film evaporation. In view of these considerations, we propose a regenerative process using falling-film evaporation within the converging–diverging tube, aiming to address the design and industrial application regarding the use of converging–diverging tubes in SO_2_ enhanced desorption of basic aluminum sulfate desulphurization–regeneration solution. Thus, to find the optimal structure of the converging–diverging tube and develop a high-efficiency falling-film evaporator, the heat and mass transfer performances of falling-film evaporation with converging–diverging tubes of different dimensions were studied. The optimal converging–diverging tube was used in falling-film evaporation desorption of the basic aluminum sulfate desulphurization–regeneration solution, and different influence factors on the desorption effect were analyzed.

## 2. Experimental method

### 2.1 Experimental apparatus

**Fig. 1:**
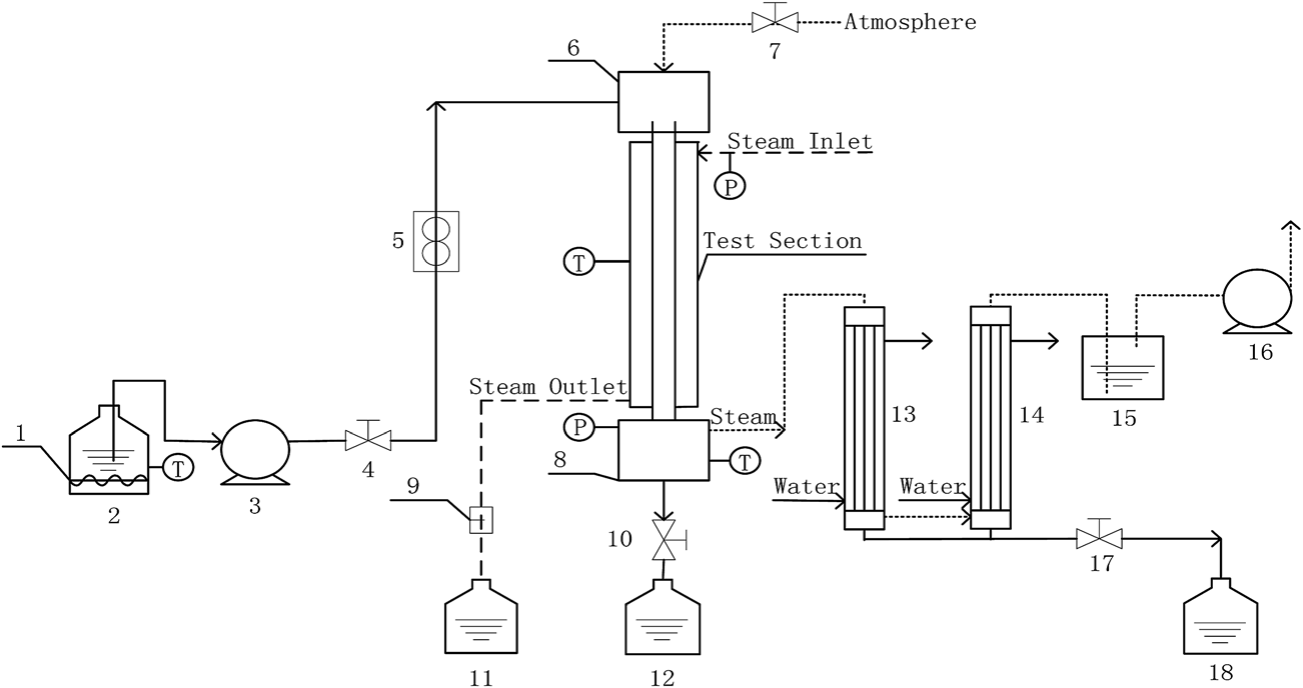
Experimental apparatus (1-auxiliary heater; 2, 11, 12, and 18-metering tanks; 3-water pump; 4, 7, 10, and 17-valves; 5-flow meter; 6-top reservoir; 8-bottom reservoir; 9-vapor–liquid separator; 13, and 14-condenser; 15-closed container; 16-vaccum pump).

**Fig. 2:**
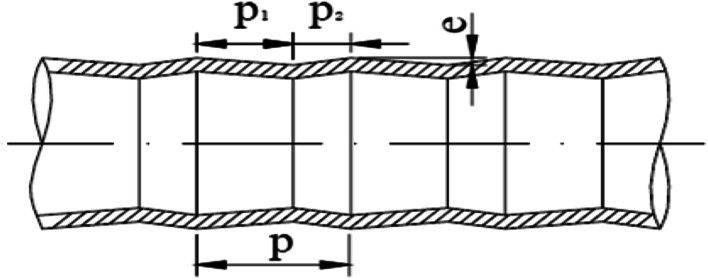
Structural scheme of converging–diverging tube.

Measurements of heat and mass transfer performances of falling-films were carried out in the experimental apparatus shown in Fig. 1. This system is primarily used to detect the heat and mass transfer performances of falling liquid films in vertical tubes. The main structure dimensions of the heat transfer tubes that contain four dimensions of converging–diverging tubes and a smooth tube are listed in Table 1. The structural scheme of the converging–diverging tubes is illustrated in Fig. 2. The fluid in the heating tank was heated by an electric heating unit to the preset temperature; then, the fluid was pumped by a water pump through a flow adjustment valve into the top water reservoir, which was connected by an adjustment valve with air. After the fluid flowed to the test section, falling liquid films were formed on the inner surface of the heat transfer tube. The vapor generated in the heat transfer tube was pumped by a vacuum pump into the condensers. Then, the condensate liquids entered a metering tank for measurement. The unevaporated liquid entered the metering tank. The outer section of the heat transfer tube was supplied with saturated steam with certain pressure and temperature. The steam-condensed, water generated during the experiments, was passed by vapor–liquid separator into a metering tank.

**Table 1:** Main structure dimension of heat transfer tubes^a^

Tube shape	Outer diameter *d*_o_ (m)	Inner diameter *d*_i_ (m)	Length of analysis segment *L* (m)	Pitch spacing/node spacing *P* (m)	Length of converging segment *P*_1_ (m)	Length of diverging segment *P*_2_ (m)	Rib height *e* (m)
C–D tube 1#	0.019	0.016	2.3	0.012	0.001	0.011	0.0006
C–D tube 2#	0.019	0.016	2.3	0.012	0.011	0.001	0.0006
C–D tube 3#	0.019	0.016	2.3	0.014	0.0105	0.0035	0.002
C–D tube 4#	0.019	0.016	2.3	0.014	0.0035	0.0105	0.002
Smooth tube	0.019	0.017	2.3	—	—	—	—

a Note: C–D: converging–diverging.

### 2.2 Experimental procedure

When the heating steam temperature outside of the heat transfer tube was constant at 373.15 K, the medium (water) was preheated to the boiling point. Then, falling liquid film experiments inside the four dimensions of converging–diverging tubes and the smooth tube were conducted by changing the water flow rate. By analyzing the heat transfer coefficient and mass transfer rate of liquid film evaporation, we determined which converging–diverging tube had the optimal heat transfer and mass transfer performances. Then, with basic aluminum sulfate desulphurization–regeneration solution as the medium, after it was preheated near the boiling point, we carried out falling-film evaporation desorption experiments inside the optimal converging–diverging tube with the smooth tube as a comparison, and the influence factors on the desorption performance were investigated. The SO_3_^2−^ concentrations before and after the desorption of desulphurization–regeneration solution were computed by the iodometric method.^30^

### 2.3 Data analysis

The peripheral flow rate of liquid films (*Γ*) and the liquid film Reynolds number (Re) inside the heat transfer tube were computed using the following equations:1
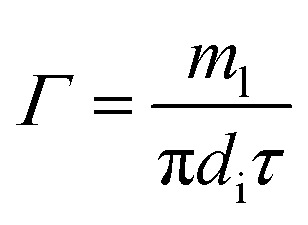
2
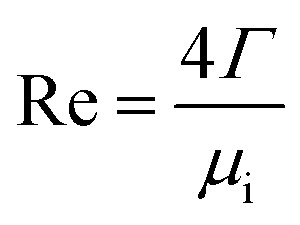
where *m*_l_ is the mass of liquid films, kg; *d*_i_ is the inside diameter of the heat transfer tube, m; *τ* is the experimental time of heat transfer in the falling-films, s; π = 3.1415926; and *μ*_i_ is the dynamic viscosity of liquid films, kg m^−1^ s^−1^.

Heat transfer was analyzed by a thermal resistance analytical method.^31^ In each experimental period, with the endothermic quantity of fluid within the heat transfer tube as the heat transfer quantity, the evaporation heat flux density of falling-films (*q*) and the total heat transfer coefficient of evaporation in falling-films (*K*_h_) were computed as follows:3
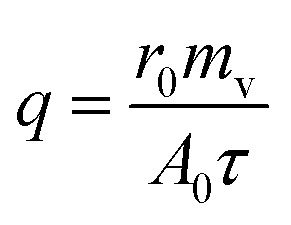
4
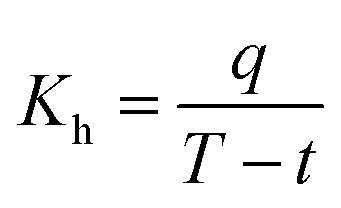
where *m*_v_ is the mass of liquid film evaporation, kg; *r*_0_ is the vaporization latent heat of liquid films under saturation temperature, kJ kg^−1^; *A*_0_ is the area of outside surface of the tube, m^2^; *T* is the heating steam temperature in the ring gap outside the tube, °C; *t* is the liquid film temperature inside the tube, °C.

The steam condensation heat transfer coefficient (*h*_o_) outside the heat transfer tube was computed by the Nusselt filmwise condensation experimental correlation:^32^5
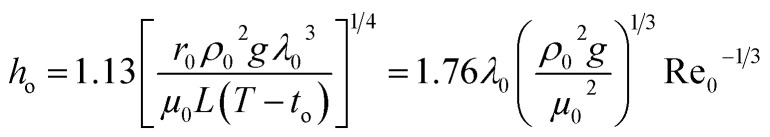
where *ρ*_0_, *λ*_0_ and *μ*_0_ are the density (kg m^−3^), thermal conductivity (W m^−1^ K^−1^) and dynamic viscosity (kg m^−1^ s^−1^) of the heating steam condensation liquid, respectively; *L* is the valid height of the vertical tube, m; *t*_o_ is the outside wall temperature, °C; *g* is the gravitational acceleration, m s^−2^; Re_0_ is the Reynolds number of the heating steam condensation liquid outside the tube.

The evaporation heat transfer coefficient of the falling-films inside the tube (*h*) was computed as follows:6
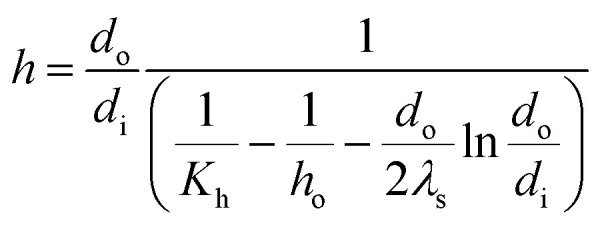
where *d*_o_, *d*_i_ and *λ*_s_ are the outside diameter (m), inside diameter (m) and thermal conductivity (W m^−1^ K^−1^) of the heat transfer tube, respectively.

The dimensionless falling-film evaporation heat transfer coefficient (*h*^+^) was computed as follows:7
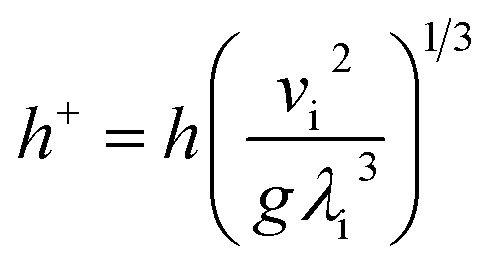
where *v*_i_ is the kinematic viscosity of liquid films (m^2^ s^−1^) and *λ*_i_ is the thermal conductivity of liquid films (W m^−1^ K^−1^).

The liquid film evaporation mass transfer rate (*u*_v_) was computed as follows:8
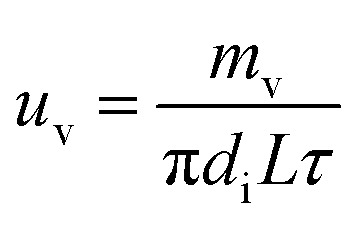


**Fig. 3:**
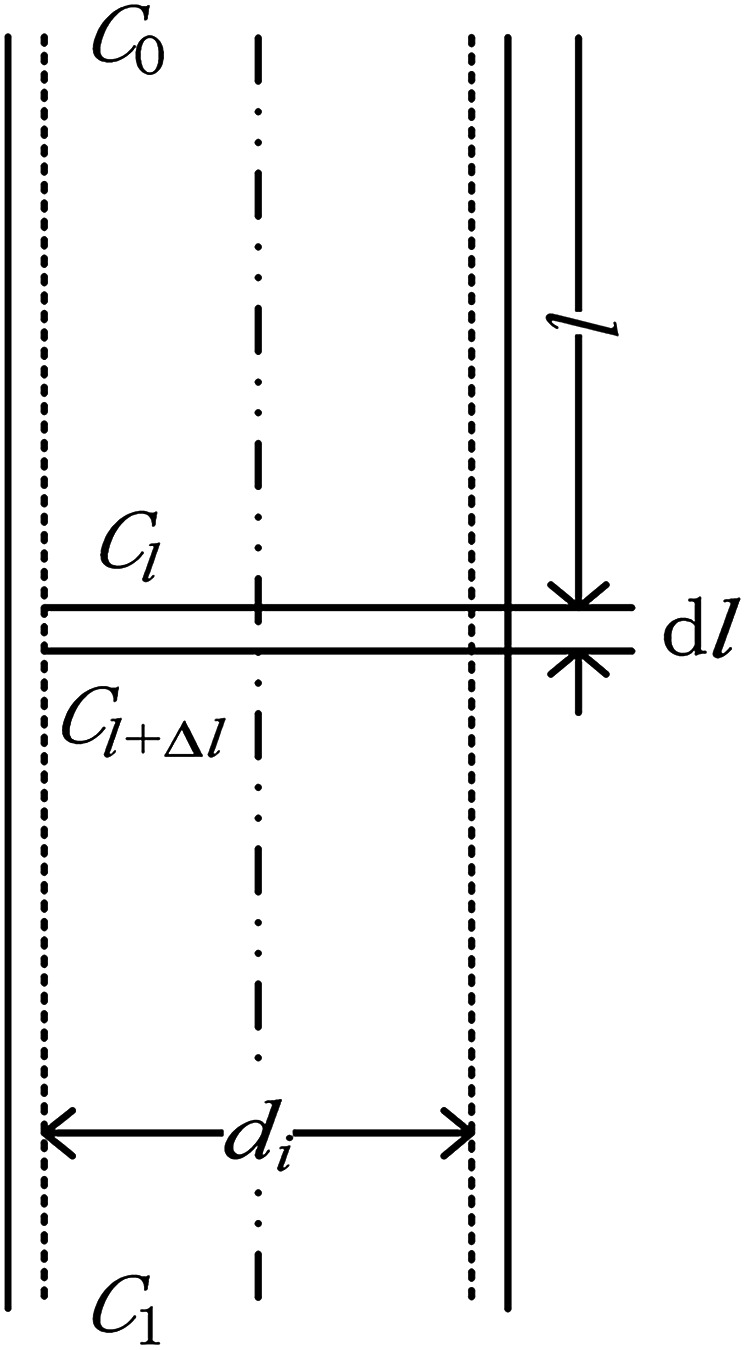
Mass transfer model of falling-film desorption.

The physical model of falling-film mass transfer is illustrated in Fig. 3. Under the stable state, the SO_2_ in the basic aluminum sulfate desulphurization–regeneration solution was continually desorbed out. Based on the SO_2_ component balance, the equation obtained was as follows:^33^

9 *U*d*C* = *N*d*A* = *K*_m_Δ*C*_m_π*d*_i_d*l*

where Δ*C*_m_ is the impetus of mass transfer.10
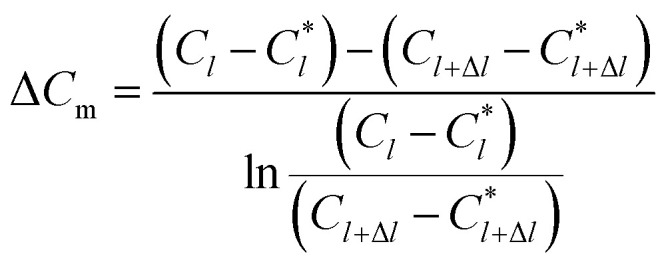
where *U* is the volumetric flow rate of liquid films, m^3^ s^−1^; *C* is the SO_3_^2−^ concentration in the liquid films, kmol m^−3^; *N* is the convection mass transfer rate of SO_2_, kmol m^−2^ s^−1^; *A* is the area of liquid films, m^2^; *K*_m_ is the total mass transfer coefficient, m s^−1^; *l* is the height of falling-films, m; *C*_*l*_ is the SO_3_^2−^ concentration at the liquid film height *l*, kmol m^−3^; C^*^_l_ is the dissolved SO_2_ concentration in solution that was balanced with the SO_2_ pressure in gas at the liquid film height *l*, kmol m^−3^; *C*_*l*+Δ*l*_ is the SO_3_^2−^ concentration at the liquid film height *l* + Δ*l*, kmol m^−3^; C^*^_l+Δ*l*_ is the dissolved SO_2_ concentration in solution that was balanced with the SO_2_ pressure in gas at the liquid film height *l* + Δ*l*, kmol m^−3^.

In the experiments, during desorption of desulphurization–regeneration solution, a larger vapor evaporation quantity indicates smaller SO_2_ concentration, which can be ignored; hence, C* approaches 0. Then, we obtain the following equation:11
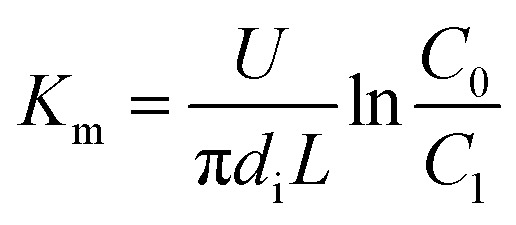
where *C*_0_ and *C*_1_ are the SO_3_^2−^ concentrations at the inlet and outlet of the liquid films, respectively, kmol m^−3^.

The dimensionless mass transfer coefficient of the falling-films (Sh) is computed as follows:12
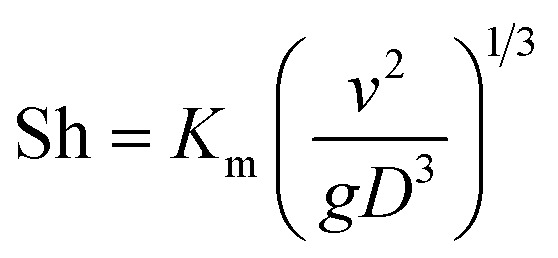
where *D* is the diffusion coefficient of SO_2_ in solution, m^2^ s^−1^.

Under the same conditions, the mass transfer coefficient at any position within the tube is considered to be the same and is equal to the total mass transfer coefficient. The SO_3_^2−^ concentration at the liquid film height *l* was computed as follows:13
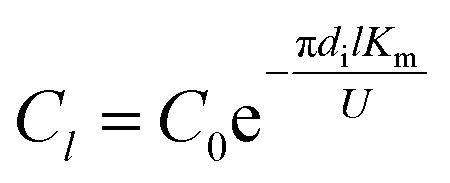


The SO_2_ desorption efficiency of desulphurization–regeneration solution (*η*) is defined as follows:14
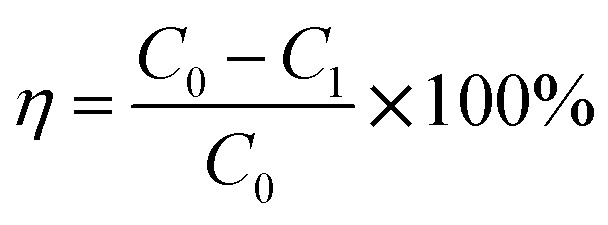


### 2.4 Uncertainty analysis

The uncertainty analysis of the experimental data was performed using the method reported by Kline *et al.*^34^ According to the uncertainty transfer and calculation method of indirect measurement, the assumption is as follows:

15 *y* = *f*(*x*_1_, *x*_2_, *x*_3_, …, *x*_*n*_)

where each variable is independent of the others, and their uncertainty is (δ*x*_1_, δ*x*_2_, δ*x*_3_…δ*x*_*n*_). The calculation formula of the relative uncertainty of indirect measurement is computed as follows:16
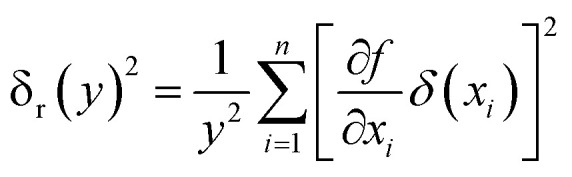


In this experiment, the volumetric flow rate of the fluid was monitored by a rotameter with the accuracy of ±1.5%, the temperature was measured by a platinum resistance temperature sensor with an accuracy of ±0.1 K, the mass was measured by pressure sensors with sensitivity of ±0.1 g, and the time was counted to the nearest 0.1 s of the stopwatch. The relative uncertainty of the evaporation heat transfer coefficient of the falling-films was obtained by combining eqn (6) and eqn (16); the evaporation mass transfer rate was obtained by combining eqn (8) and eqn (16); the mass transfer coefficient was obtained by combining eqn (11) and eqn (16). Through uncertainty propagation analysis, the maximum uncertainties of the heat transfer coefficient, mass transfer rate and mass transfer coefficient in the experiments were computed to be 6.71%, 2.0% and 8.18%, respectively.

## 3. Results and discussion

### 3.1 Heat and mass transfer performances with converging–diverging tubes of different dimensions

**Fig. 4:**
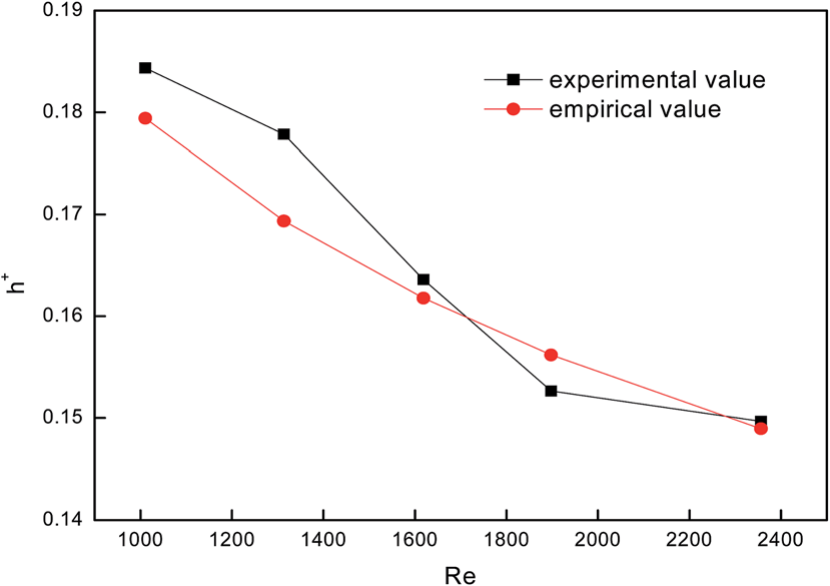
Comparison between falling-film evaporation and the empirical formula.

To test the accuracy of the experimental system, we used a smooth tube as the control, and validated the reliability of the system by comparing with previous experimental results. Fig. 4 shows a comparison between falling-film evaporation and the Chun & Seban empirical formula^35^ with the largest error below ±6%. The experimental results of falling-film evaporation based on the system are consistent with the previous findings, indicating that this system is highly reliable.

**Fig. 5:**
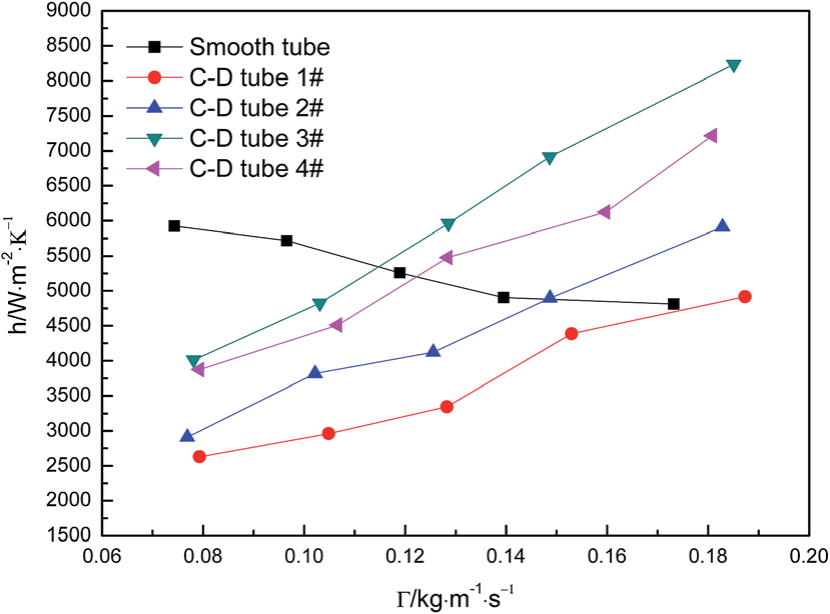
Relationship between the evaporation heat transfer coefficient and the liquid film flow rate.

Fig. 5 shows the curves of evaporation heat transfer coefficients of falling-films along with the liquid film flow rate of 0.07–0.18 kg m^−1^ s^−1^ for the four converging–diverging tubes and the smooth tube. Clearly, with an increase in the flow rate, the falling-film evaporation heat transfer coefficients inside all the converging–diverging tubes increase. Compared with the smooth tube, converging–diverging tubes exhibited good heat transfer performance in the range of flow rate of 0.12–0.18 kg m^−1^ s^−1^. This indicates that the converging–diverging tubes are appropriate for falling-film evaporation with large liquid film flow rate, while the smooth tube is better for falling-film evaporation with small liquid film flow rate. This is because for a small liquid film Reynolds number, the film thickness plays a dominant effect on the evaporation heat transfer coefficients of falling-films. The converging–diverging tube has a periodic alternation of converging segments and diverging segments, which leads to the periodical “increase and decrease” of film thickness during the falling-film process, but the average film thickness is larger than that of the smooth tube with constant film thickness, so the heat transfer performance is weakened. As the liquid film Reynolds number increases, the turbulence of liquid films in the converging–diverging tubes is intensified, so the role of turbulence-induced heat transfer surpasses that of the film thickness and the falling-film evaporation heat transfer coefficient gradually increases. As for different dimensions, the falling-film evaporation heat transfer coefficients of both converging–diverging tubes 3# and 4# are better than tubes 1# or 2#. This is because tubes 3# and 4# have larger rib heights, which help to efficiently induce the disturbance of liquid films.^36^ Moreover, the falling-film evaporation heat transfer coefficients of converging–diverging tubes 3# and 2# are better than tubes 4# and 1#, respectively. This is primarily because the heat transfer performance is enhanced in the converging segment and weakened in the diverging segment according to field synergy theory. Thus, at the same rib height and rib pitch, the longer the converging segment of the converging–diverging tube is, the better the heat transfer performance is.^37,38^ At the liquid film flow rate of 0.17 kg m^−1^ s^−1^, tube 3# has an evaporation heat transfer coefficient 1.6 times larger than that of the smooth tube.

**Fig. 6:**
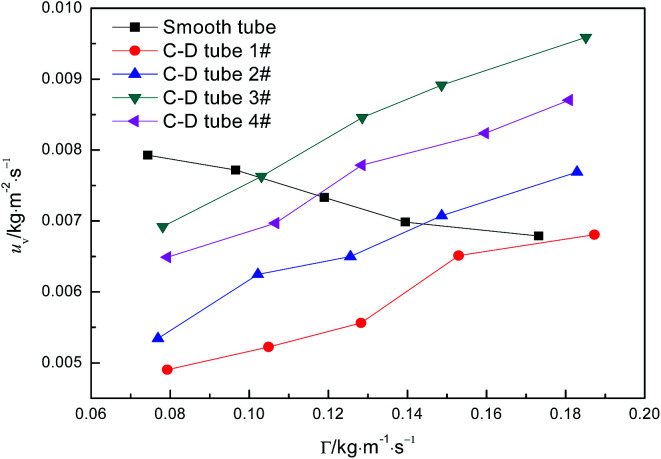
Effect of the perimeter flow rate on falling-film evaporation mass transfer rate.

Fig. 6 shows the relationship curves between the perimeter flow rate and the evaporation mass transfer rate of falling-films for the four converging–diverging tubes and the smooth tube. The evaporation mass transfer rates of falling-films inside all the converging–diverging tubes increase with an increase in the perimeter flow rate of the liquid films. This is primarily because the evaporation mass transfer rate of falling-films is largely associated with the evaporation heat transfer coefficient as a larger evaporation heat transfer coefficient promotes heat absorption by liquid films, leading to the increase in evaporation of the liquid films and thus the evaporation mass transfer rate. Moreover, with the increase in flow rate, the evaporation mass transfer rates of both tubes 3# and 4# surpass those of tubes 1# and 2# or the smooth tube; thus, tubes 3# and 2# are better than tubes 4# and 1#, respectively. When the perimeter flow rate of liquid films is 0.173 kg m^−1^ s^−1^, the evaporation mass transfer rate of the falling-films in tube 3# is 0.0094 kg m^−2^ s^−1^, which is 1.38 times larger than the smooth tube. Thus, according to the comparative analysis of the heat and mass transfer performances inside the four converging–diverging tubes, converging–diverging tube 3# is optimal.

### 3.2 Desorption effect inside the optimal converging–diverging tube

#### 3.2.1 Effect of the different flow rates

**Fig. 7:**
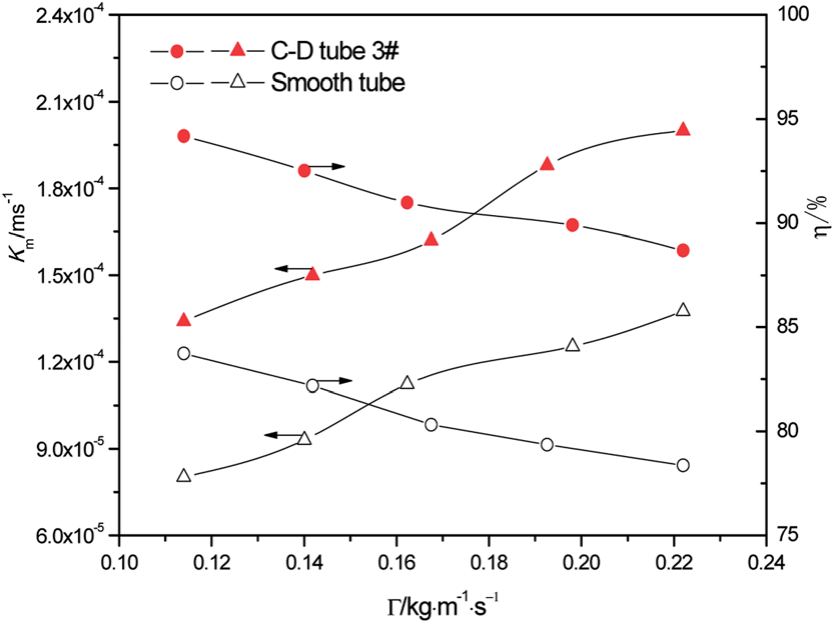
Effect of the liquid film perimeter flow rate on the mass transfer coefficient and SO_2_ desorption efficiency.

At the heating temperature of 381.15 K, sulfur concentration of 0.06 kmol m^−3^, aluminum concentration of 20 kg m^−3^ and basicity of 20%, the relationship between the average mass transfer coefficient and liquid film flow rate in the basic aluminum sulfate desulphurization–regeneration solution is illustrated in Fig. 7. With the rise in flow rate, the falling-film average mass transfer coefficients of the converging–diverging tube 3# and the smooth tube both increase. Under the same conditions, the falling-film average mass transfer coefficient inside the converging–diverging tube is 44–67% higher than the smooth tube. The main reason is that the converging–diverging tube promotes the fluid disturbance near the wall, enhances turbulence and reduces the thickness of the viscous bottom layer. During the falling-film desorption of basic aluminum sulfate desulphurization–regeneration solution, SO_2_ desorption efficiency gradually decreases with the liquid film perimeter flow rate (Fig. 7). At the same inlet sulfur concentration, as the flow rate increases, the outlet sulfur concentrations inside the converging–diverging tube 3# and the smooth tube gradually become higher. This is primarily because besides the mass transfer coefficient, the falling-film desorption is also correlated with the flow rate. It is positively correlated with the mass transfer coefficient and negatively correlated with flow rate. Thus with an increase in the flow rate, though the mass transfer coefficient is improved, the effect of flow rate surpasses that of the mass transfer coefficient, weakening the desorption. Under the same flow rate, the outlet sulfur concentration of the falling-films in the converging–diverging tube is lower than that in the smooth tube. At the liquid film perimeter flow rate of 0.114 kg m^−1^ s^−1^, the outlet sulfur concentration is 64% lower and the desorption efficiency (up to 94.2%) is 10.5% higher in the converging–diverging tube than in the smooth tube. At the flow rate of 0.222 kg m^−1^ s^−1^, the sulfur concentration is 48% lower and the desorption efficiency (up to 88.7%) is 10.3% higher in the converging–diverging tube than in the smooth tube. Moreover, when the desulphurization–regeneration solution flows along the tube length, the sulfur concentrations first decline rapidly and then slowly, indicating that the higher sulfur concentrations contribute to desorption.

#### 3.2.2 Effect of the different sulfur concentrations

**Fig. 8:**
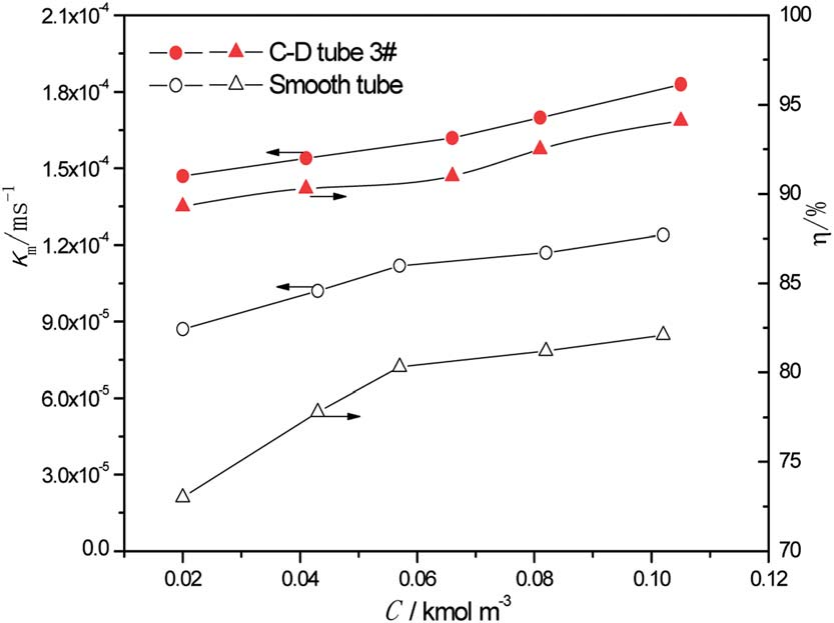
Effect of the inlet sulfur concentration on the mass transfer coefficient and SO_2_ desorption efficiency.

At the heating temperature of 381.15 K, perimeter flow rate of 0.162 kg m^−1^ s^−1^, aluminum concentration of 20 kg m^−3^ and basicity of 20%, the relationship between the average mass transfer coefficient and inlet sulfur concentration in basic aluminum sulfate desulphurization–regeneration solution is illustrated in Fig. 8. With the rise in inlet sulfur concentration, the falling-film average mass transfer coefficients of both the converging–diverging tube 3# and the smooth tube increase. This is primarily because the mass transfer coefficient is correlated with the sulfur concentration gradient at the film thickness direction of the desulphurization–regeneration solution according to film theory. At the same flow rate, as the inlet sulfur concentration increases, the concentration gradient at the film thickness direction rises. Thus, the mass transfer coefficient increases for both tubes, but the falling-film average mass transfer coefficient inside the converging–diverging tube 3# is 44–69% higher than that in the smooth tube. During the falling-film desorption of basic aluminum sulfate desulphurization–regeneration solution, SO_2_ desorption efficiency gradually increases with the inlet sulfur concentration (Fig. 8). In comparison, at the inlet sulfur concentration of 0.02 kmol m^−3^, the outlet sulfur concentration is 61% lower and the desorption efficiency (up to 89.3%) is 16.3% higher in the converging–diverging tube than in the smooth tube. At the inlet sulfur concentration of 0.1 kmol m^−3^, the sulfur concentration is 67% lower and the desorption efficiency (up to 94.1%) is 12.0% higher in the converging–diverging tube than in the smooth tube.

#### 3.2.3 Effect of the different heating temperatures

**Fig. 9:**
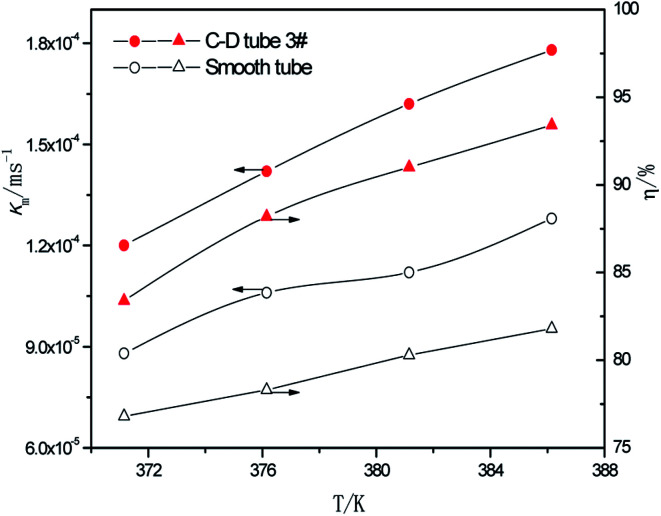
Effect of the heating temperature on the mass transfer coefficient and SO_2_ desorption efficiency.

At the perimeter flow rate of 0.162 kg m^−1^ s^−1^, sulfur concentration of 0.06 kmol m^−3^, aluminum concentration of 20 kg m^−3^ and basicity of 20%, the relationship between the average mass transfer coefficient and heating temperature is illustrated in Fig. 9. With the rise of heating temperature, the falling-film average mass transfer coefficients of both the converging–diverging tube 3# and the smooth tube increase. This is primarily because the mass transfer coefficient is also correlated with SO_2_ diffusion coefficient of the desulphurization–regeneration solution according to film theory, and the diffusion coefficient is proportional to the desulphurization–regeneration solution temperature. With the rise of heating temperature, the desulphurization–regeneration solution temperature increases. Thus, the mass transfer coefficient increases for both tubes, but the falling-film mass transfer coefficient inside the converging–diverging tube is 33–44% higher than in the smooth tube. During the falling-film desorption of basic aluminum sulfate desulphurization–regeneration solution, SO_2_ desorption efficiency gradually increases with the heating temperature (Fig. 9). At the same flow rate and inlet sulfur concentration, as the heating temperature rises, the outlet sulfur concentrations in the converging–diverging tube 3# and the smooth tube gradually drop. At the heating temperature of 371.15 K, the outlet sulfur concentration is 29% lower and the desorption efficiency (up to 83.4%) is 6.7% higher in the converging–diverging tube than in the smooth tube. At the heating temperature of 386.15 K, the outlet sulfur concentration is 63% lower and the desorption efficiency (up to 93.4%) is 11.5% higher in the converging–diverging tube than in the smooth tube.

#### 3.2.4 Correlation derived from the data

As discussed above and shown in Fig. 7–9, the mass transfer coefficient of the falling-film evaporation for the converging–diverging tube 3# are higher than that for the smooth tube. Thus, the correlation should be used to predict the mass transfer coefficients of falling-film evaporation in the converging–diverging tube 3#. For engineering purposes, we tried to model *K*_m_ in functions of important influencing parameters only. The Sherwood numbers and SO_2_ desorption efficiency of the falling-film evaporation inside the converging–diverging tube 3# and smooth tube are calculated as follows:

For the converging–diverging tube 3#

17 Sh = 2.0 × 10^−3^ Re^0.604^ Sc^0.44^

18 *η* = [1 − exp(−2.0 × 10^−3^ Re^0.604^ Sc^0.44^ *g*^1/3^*v*^−2/3^DAU^−1^)] × 100%

For the smooth tube

19 Sh = 1.0 × 10^−4^ Re^0.935^ Sc^0.44^

20 *η* = [1 − exp(−1.0 × 10^−4^ Re^0.935^ Sc^0.44^ *g*^1/3^*v*^−2/3^DAU^−1^)] × 100%

**Fig. 10:**
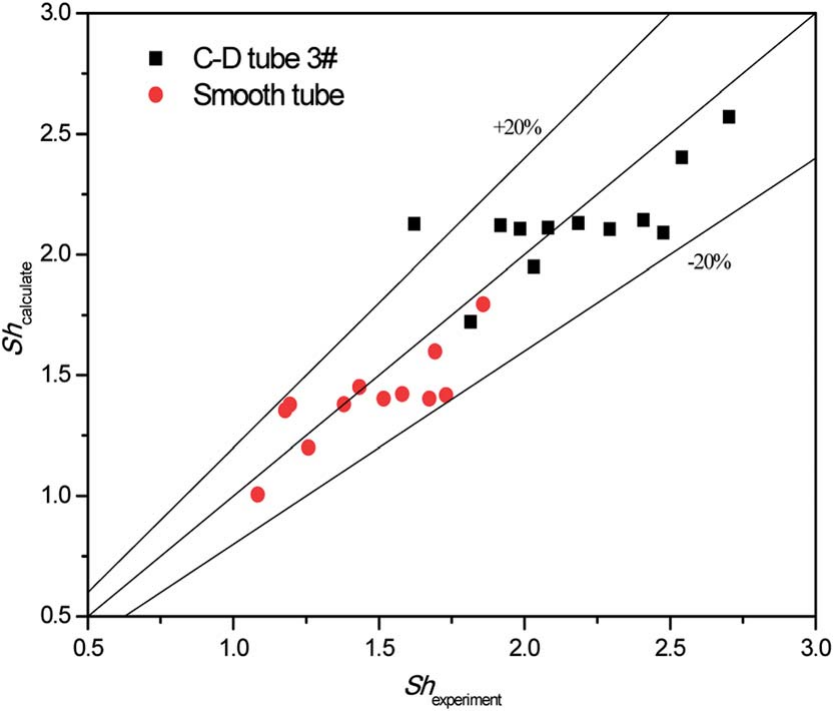
Comparison of the experimental data and the calculated value on mass transfer coefficient.

**Fig. 11:**
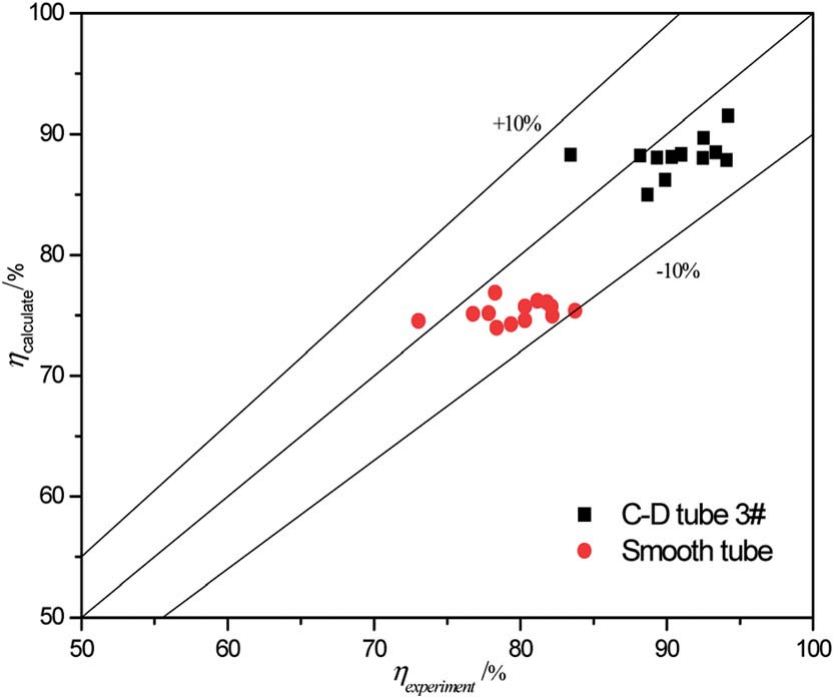
Comparison of the experimental data and the calculated value on SO_2_ desorption efficiency.

The validity of using eqn (17)–(20) to predict the experimental mass transfer coefficient and SO_2_ desorption efficiency are shown in Fig. 10 and 11, respectively. As shown in Fig. 10, for the correlation on the mass transfer coefficients inside the converging–diverging tube, 93% of the data falls within ±20% error; for the smooth tube, 100% are within ±20% error. As shown in Fig. 11, for the correlation on the SO_2_ desorption efficiency inside the converging–diverging tube and smooth tube, 100% of the data falls within ±10% error. Overall, good agreement has been observed between experimental data and theoretical prediction.

## 4. Conclusions

We developed a new type of falling-film evaporator for SO_2_ enhanced desorption experiments. To find the optimal structure of the converging–diverging tube and develop a high-efficiency falling-film evaporator, the heat and mass transfer performances of converging–diverging tubes with different dimensions were studied. It was found that converging–diverging tubes with large liquid film flow rate performed well in the falling-film evaporation, and their rib heights largely affected the heat and mass transfer performances. At the same rib height and rib pitch, the longer the converging segment of the converging–diverging tube was, the better the heat transfer performance was. The evaporation heat transfer coefficient and evaporation mass transfer rate in the optimal converging–diverging tube were 1.6 and 1.38 times larger than the smooth tube, respectively. The optimal converging–diverging tube was used in the falling-film desorption of desulphurization–regeneration solution: the mass transfer coefficient increased and SO_2_ desorption efficiency decreased with an increase in the flow rate, but both increased with an increase in sulfur concentration or heating temperature. Smaller flow rate, higher sulfur concentration, and higher heating temperature were more constructive to SO_2_ desorption. The mass transfer coefficient in the converging–diverging tube was 33–69% higher than that in the smooth tube, and thus the SO_2_ desorption efficiency was greatly improved. At the perimeter flow rate of 0.114–0.222 kg m^−1^ s^−1^, the desorption efficiency in the converging–diverging tube was up to 94.2% and was 10.3–10.5% higher than that in the smooth tube. At the inlet sulfur concentration of 0.02–0.1 kmol m^−3^, the desorption efficiency was up to 94.1% and was 12.0–16.3% larger than that in the smooth tube. At the heating temperature of 371.15–386.15 K, the desorption efficiency was up to 93.4% and was 6.7–11.5% larger than that in the smooth tube. Moreover, correlations were obtained to predict the mass transfer coefficient and SO_2_ desorption efficiency. This study forms a basis for the process design and industrial application of converging–diverging tubes into a new type of falling-film evaporator for SO_2_ desorption of basic aluminum sulfate desulphurization–regeneration solution.

## Conflicts of interest

The authors declare no competing financial interest.

## Nomenclature

*A*_0_
: Area of outside surface of the tube, m^2^
*A*
: Area of liquid films, m^2^
*C*
: SO_3_^2−^ concentration in the liquid films, kmol m^−3^
C*: Dissolved SO_2_ concentration in solution that was balanced with the SO_2_ pressure in gas, kmol m^−3^
*C*_0_
: SO_3_^2−^ concentrations at the inlet of the liquid films, kmol m^−3^
*C*_1_
: SO_3_^2−^ concentrations at the outlet of the liquid films, kmol m^−3^
*C*_*l*_
: SO_3_^2−^ concentration at the liquid film height *l*, kmol m^−3^
*C* ^*^_l_
: Dissolved SO_2_ concentration in water that was balanced with the SO_2_ pressure in gas at the liquid film height *l*, kmol m^−3^
*C*_*l*+Δ*l*_
: SO_3_^2−^ concentration at the liquid film height *l* + Δ*l*, kmol m^−3^
*C* ^*^_l+Δ*l*_
: Dissolved SO_2_ concentration in water that was balanced with the SO_2_ pressure in gas at the liquid film height *l* + Δ*l*, kmol m^−3^
Δ*C*_m_: Impetus of mass transfer, kmol m^−3^
*D*
: Diffusion coefficient of SO_2_ in solution, m^2^ s^−1^
*d*_o_
: Outside diameter of the heat transfer tube, m
*d*_i_
: Inside diameter of the heat transfer tube, m
*g*
: Gravitational acceleration, m s^−2^
*h*_o_
: Steam condensation heat transfer coefficient outside the tube, W m^−2^ K^−1^
*h*
: Evaporation heat transfer coefficient of the falling film inside the tube, W m^−2^ K^−1^
*h* ^+^
: Dimensionless falling-film evaporation heat transfer coefficient
*K*_h_
: Total heat transfer coefficient of evaporation in falling-films, W m^−2^ K^−1^
*K*_m_
: Total mass transfer coefficient, m s^−1^
*L*
: Valid height of the vertical tube, m
*l*
: Height of falling-films, m
*m*_l_
: Mass of liquid films, kg
*m*_v_
: Mass of liquid film evaporation, kg
*N*
: Convection mass transfer rate of SO_2_, kmol m^−2^ s^−1^
*q*
: Evaporation heat flux density of falling-films, W m^−2^
Re: Liquid film Reynolds number inside the heat transfer tube
Re_0_: Reynolds number of the heating steam condensation liquid outside the tube
*r*_0_
: Vaporization latent heat of liquid films under saturation temperature, kJ kg^−1^
Sh: Dimensionless mass transfer coefficient of the falling-films
*T*
: Heating steam temperature in the ring gap outside the tube, K
*t*
: Liquid film temperature inside the tube, K
*t*_o_
: Outside wall temperature, K
*U*
: Volumetric flow rate of the liquid films, m^3^ s^−1^
*u*_v_
: Liquid film evaporation mass transfer rate, kg m^−2^ s^−1^

## Greek symbols

*Γ*
: Peripheral flow rate of liquid films inside the tube, kg m^−1^ s^−1^
*η*
: SO_2_ desorption efficiency, %
*λ*_i_
: Thermal conductivity of liquid films, W m^−1^ K^−1^
*λ*_0_
: Thermal conductivity of the heating steam condensation liquid, W m^−1^ K^−1^
*λ*_s_
: Thermal conductivity of the heat transfer tube, W m^−1^ K^−1^
*μ*_i_
: Dynamic viscosity of liquid films, kg m^−1^ s^−1^
*μ*_0_
: Dynamic viscosity of the heating steam condensation liquid, kg m^−1^ s^−1^
*v*_i_
: Kinematic viscosity of liquid films, m^2^ s^−1^
π: 3.1415926
*ρ*_0_
: Density of the heating steam condensation liquid, kg m^−3^
*τ*
: Experimental time of heat transfer in the falling-films, s

## Supplementary Material
